# Ocular Syphilis and Syphilitic Meningitis as the Initial Symptoms of Neurosyphilis in an HIV-Negative Patient: A Case Report

**DOI:** 10.7759/cureus.57675

**Published:** 2024-04-05

**Authors:** Papul Chalia, Zekeil Factor, Mansoureh Mamarabadi

**Affiliations:** 1 Neurology, Penn State Health Milton S. Hershey Medical Center, Hershey, USA; 2 Neurology, Penn State College of Medicine, Hershey, USA

**Keywords:** syphilis screening, visual disturbances, syphilitic meningitis, ocular syphilis, neurosyphilis

## Abstract

Syphilis is an infectious disease caused by the spirochete bacteria *Treponema pallidum* and is most commonly transmitted via contact of mucous membranes with infectious lesions during sexual intercourse. It is called the "great mimicker" due to its ability to infect a wide variety of organs and, as a result, produce a multitude of symptoms. Neurosyphilis, an infection of the central nervous system, can occur at any stage of infection. Cases of early neurosyphilis may not present with any prior history of syphilis infection or classical symptoms of primary or secondary infection. Homosexual men are disproportionately affected by the increasing rate of transmission.In this case, a 43-year-old man was diagnosed with neurosyphilis, initially presenting with bilateral papilledema concerning for idiopathic intracranial hypertension. A detailed social history revealed that the individual was sexually active with a male partner. Despite nonreactive results from the rapid plasma reagin and CSF Venereal Disease Research Laboratory tests, further serum workup yielded positive results for treponemal antibodies. Evidence of facial nerve involvement was also found on MRI. These findings were consistent with a diagnosis of ocular syphilis with syphilitic meningitis involving cranial nerve VII. This case demonstrates the importance of clinical suspicion for syphilis when indicated by social history, even when screening tests are negative, due to the potential for false negatives and highly variable clinical presentation.

## Introduction

The incidence of syphilis, at all stages, has been increasing since 2000. The course of infection can classically be divided into four stages: primary syphilis due to local infection; secondary syphilis due to bacteremia; latent syphilis, which is asymptomatic; and tertiary syphilis, which often occurs decades after initial infection and causes organ damage, most commonly in the nervous and cardiovascular systems. However, unlike most kinds of organ damage, neurosyphilis can occur at any stage of infection, although commonly targeted areas for invasion within the central nervous system and resultant clinical manifestations can vary depending on the stage [1]. In 2020, the Centers for Disease Control and Prevention reported 133,945 cases of syphilis, including 41,655 cases of primary syphilis [2].

Neurosyphilis is rare; of the reported incidence of syphilis, only 3-5% of patients will go on to develop neurosyphilis [3]. The invention of antibiotics has greatly reduced the percentage of patients who progress to neurosyphilis, and currently, the majority of patients who develop neurosyphilis are those who are co-infected with human immunodeficiency virus (HIV) [4,5]. Of note, asymptomatic neuro-invasion is believed to occur very early in the course of infection in all patients but is more likely to progress to symptomatic neurosyphilis when patients fail to clear the bacteria from the nervous system under immunocompromised condition [2]. Patients with early neurosyphilis that have symptoms tend to have findings of meningitis or cranial nerve involvement, predominantly II, VI, and VIII [1].

Throughout the 1990s, the incidence of syphilis had declined to the point of near elimination, in tandem with advances in awareness and treatment of the HIV epidemic. But since the early 2000s, there has been an ongoing rise in the incidence of syphilis, which disproportionately affects men who have sex with men (MSM), who make up 82% of men infected with syphilis [6,7]. Clinicians should recognize that the incidence of syphilis has been increasing, especially among MSM, including those without HIV co-infection.

## Case presentation

A 43-year-old Caucasian man with a history of migraine headaches developed bilateral visual disturbances, which he described as “radioactive signs spinning like fan blades.” Approximately six weeks before seeking medical attention, he began experiencing fever, chills, and myalgias, for which he was prescribed a course of oral cephalexin that he did not complete, and these symptoms resolved after three weeks. Three weeks prior to his presentation, he began to experience visual disturbances that initially manifested, as well as recurring headaches with pain behind his left eye, both predominantly occurring early in the morning and interrupting his sleep. These headaches persisted for three weeks leading up to his emergency department (ED) visit.

Four days before seeking medical help, the patient noticed “a fog” over his left eye, a black structure in the periphery of his left eye, and a black oval-shaped structure in the center of his right eye. The following morning, he complained of left-sided ear fullness along with worsening vision, prompting his visit to an outside ED. A comprehensive stroke workup, including, computed tomography (CT) of the head, CT angiography (CTA) of the head and neck, magnetic resonance imaging (MRI) without contrast, and an echocardiogram, revealed no remarkable findings. Complete blood count, comprehensive metabolic panel, and urine analysis studies were also unremarkable. The patient was discharged with the plan of outpatient ophthalmology evaluation.

Outpatient eye examination revealed the following: visual acuity 20/40 in the left eye and 20/30 in the right eye, with a color vision of 11/11 on Ishihara color plates bilaterally. Pupils were equal, without any afferent pupillary defect. Extraocular movements were intact. The left optic nerve showed a blurred disc margin with obscuration of vessels at the margin and flame hemorrhages at the disc, and the right optic nerve showed inferotemporal disc edema. Tortuous vessels were noted bilaterally. He was referred to our hospital for a workup of bilateral papilledema. At the ED, vitals were within normal limits, and neurological examination was normal except for bilateral papilledema and reduced hearing to finger friction rub in the left ear. Initial lab work showed a normal complete blood count, comprehensive metabolic panel, erythrocyte sedimentation rate, and C-reactive protein.

**Figure 1:**
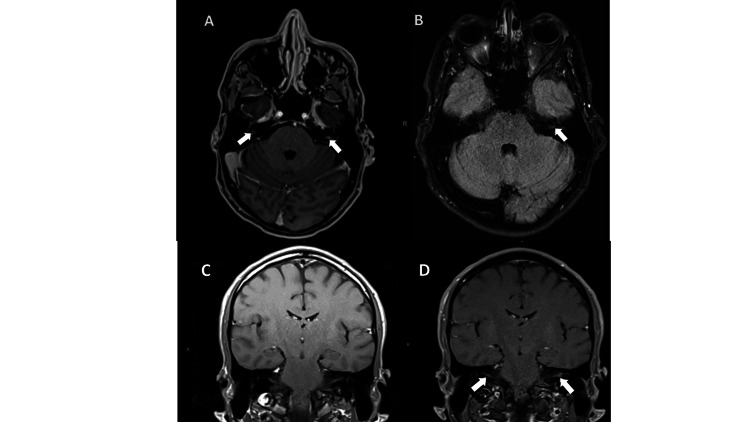
Axial post-contrast T1 (A), axial FLAIR (B), coronal pre-contrast T1 (C), coronal post-contrast T1, and (D) brain magnetic resonance imaging The image shows enhanced linear areas within the internal auditory canals bilaterally, with the left side exhibiting more enhancement than the right, specifically along the intracanalicular segment of the facial nerves, accompanied by a corresponding FLAIR hyperintense signal (white arrow). FLAIR, fluid-attenuated inversion recovery.

The patient was hospitalized for assessment of possible intracranial hypertension and venous sinus thrombosis. Brain MRI revealed abnormal linear areas of enhancement in the internal auditory canals bilaterally, with the left side showing more enhancement than the right (Figure 1). Magnetic resonance angiography (MRA) and magnetic resonance venography (MRV) were unremarkable. A lumbar puncture revealed an opening pressure of 10 cm H2O, which did not confirm the presence of intracranial hypertension. Two days later, ophthalmology evaluation revealed 360° disc edema with obscuration of vessels and flame hemorrhages at disc bilaterally, with optic nerve swelling more pronounced on the left, and intraretinal hemorrhage throughout the left fovea.

Lumbar puncture analysis revealed an elevated nucleated cell count of 47/μl (lymphocytic predominant), elevated protein of 52 mg/dL, and normal glucose level. These findings were inconclusive, so a more in-depth social history was taken, which found that the patient had a male life partner with whom he was sexually active. Further serum workup discovered positive treponemal antibody screen, fluorescent treponemal antibody absorption (FTA-ABS), and *Treponema pallidum* antibody particle agglutination (TPPA). Both rapid plasma reagin (RPR) and cerebrospinal fluid Venereal Disease Research Laboratory (CSF-VDRL) tests were negative, as was serum HIV antibody testing. The immunology workup was negative for chlamydia, *Cryptococcus*, gonorrhea, and Lyme antibodies. Further investigations of the patient's CSF, such as flow cytometry, cytology, polymerase chain reaction (PCR) for herpes simplex virus 1 and 2 and varicella-zoster virus, tuberculosis testing (PCR, culture, and Ziehl-Neelsen stain), India ink for *Cryptococcus*, gram stain, and culture, were all negative (Table 1). CT scans of the abdomen, pelvis, and thorax with contrast were negative for malignancy or sarcoidosis. Based on the investigations, a diagnosis of early neurosyphilis was made, presenting as ocular syphilis with meningitis involving cranial nerve VII.

**Table 1:** The results of the serum and cerebral spinal fluid studies in the presented case with neurosyphilis ALT, alanine transaminase; AST, aspartate aminotransferase; BUN, blood urea nitrogen; CSF, cerebral spinal fluid; FTA-ABS, fluorescent treponemal antibody absorption; HIV, human insufficiency virus; HSV, herpes simplex virus; PCR, polymerase chain reaction; RBC, red blood cell; VDRL, Venereal Disease Research Laboratory test; VZV, varicella-zoster virus; WBC, white blood cell.

Measurement	Result	Reference range
WBC count	7.50	4-10.4 K/uL
Hemoglobin	13.9	13-17 g/dL
RBC count	4.3	4.10-5.60 M uL
Platelet count	191	150-350 k/uL
Sodium	143	136-145 mmol/L
Potassium	3.8	3.5-5.1 mmol/L
Chloride	102	98-107 mmol/L
BUN	11	6-23 mg/dL
Creatinine	1.01	0.70-1.130 mg/dL
Glucose	103	74-109 mg/dL
Calcium	9.4	8.4-10.2 mg/dL
AST	21	0-40 unit/L
ALT	22	0-41 unit/L
C-reactive protein	<0.30	<0.50 mg/dL
Erythrocyte sedimentation rate	13	0-25 mm/hr
Chlamydia trachomatis, by PCR	Negative	Negative
Cryptococcal antigen	Negative	Negative
Neisseria gonorrhoeae, by PCR	Negative	Negative
Lyme antibodies, IgG/IgM	Negative	Negative
T-Spot tuberculosis test	Negative	Negative
Rapid plasma reagin	Non-reactive	Non-reactive
Treponema pallidum antibody (particle agglutination)	Reactive	Non-reactive
FTA-ABS	Reactive	Non-reactive
HIV antigen-antibody screening	Non-reactive	Non-reactive
Color, CSF	Colorless	Colorless
Opening pressure	10	6-25 cmH2O
Nucleated cell, CSF	42	0 to 5 cells/uL
RBC, CSF	2	0 cells/microL
Neutrophil, CSF	4	0%-6%
Lymphocyte, CSF	85	40%-80%
Basophil, CSF	0	1-3%
Eosinophil, CSF	0	1-4%
Monocyte, CSF	11	15-45%
Protein, CSF	52	15-45 mg/dL
VDRL, CSF	Non-reactive	Non-reactive
HSV 1 and 2, CSF by PCR	Negative	Negative
VZV, CSF by PCR	Negative	Negative
Tuberculosis, CSF by PCR, culture	Negative	Negative
Flow cytometry	Negative	Negative
Cytology	Negative	Negative
Cryptococcus	Negative	Negative

**Figure 2:**
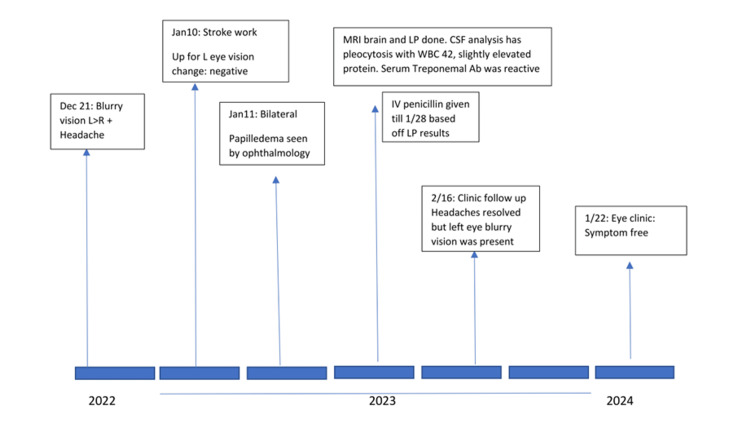
Timeline of disease course from onset of symptoms to resolution LP, lumbar puncture.

The patient was treated with three weeks of IV penicillin, as well as indomethacin for the management of headaches, both of which were continued following his discharge from the hospital after a one-week admission. At follow-up four weeks post-discharge, the patient’s bilateral papilledema had resolved, although optic disc blurring was still present. Hearing acuity was intact to finger rub bilaterally. After a year, the patient reported his vision had fully returned to normal and his headaches had resolved and stated that he would be more cautious about his sexual behavior. The course of his symptom progression and recovery is outlined below (Figure 2).

## Discussion

We present the case of a young man with acute blurred vision, which was initially thought to be idiopathic intracranial hypertension, but progression in visual symptoms after a week led to further investigation. Ocular symptoms are very rare in neurosyphilis and ocular syphilis accounts for only 1-5% of neurosyphilis cases in the United States [8]. However, optic nerve involvement occurs in approximately half of ocular syphilis cases, so cranial nerve involvement is an important pathway to consider in cases of suspected syphilis with ocular symptoms, even in the absence of other neurologic symptoms [9]. The diagnosis of neurosyphilis is a challenge as it can present with variable clinical signs, and cannot be excluded on the basis of a negative CSF-VDRL test [10]. Similarly, RPR, which also quantifies the amount of serum anticardiolipin antibodies, may also return a false negative; these tests are less sensitive in earlier stages of infection, particularly when serum antibody levels are extremely high [11,12]. Furthermore, tests that are nonspecific to *Treponema pallidum* have a high false positive rate, as anticardiolipin antibodies may be present due to a wide array of other infections, vaccinations, or autoimmune diseases, or in pregnancy or old age [11,12]. *Treponema pallidum*-specific tests such as FTA-ABS, TPPA, and *Treponema pallidum* hemagglutination assay (TPHA) can also be nonspecific [13]. In addition, while these tests may detect infection earlier than anticardiolipin antibody testing, they are still less sensitive in cases of primary syphilis [12,13]. This should be taken into consideration as neurosyphilis can occur even at the early stages of infection.

In this case, the patient presented with headaches and blurred vision, which could mislead the diagnosis and prompt the workup toward space-occupying lesions, vascular diseases, autoimmune or inflammatory conditions, and intracranial hypertension rather than syphilis infection; therefore, clinical suspicion plays a major role in determining the course of the investigation. While ocular syphilis is rare overall, decreased visual acuity is one of its most common presentations, with many also experiencing ocular pain, both of which were present in this patient. Ocular findings with papilledema, optic neuritis, and uveitis are all consistent with ocular manifestations of neurosyphilis, with bilateral ocular involvement occurring in the majority of cases [9,14]. Acute onset of these visual symptoms with supporting ocular findings and risk factors in social history should increase clinical suspicion for ocular syphilis.

In addition, MRI findings showed increased bilateral enhancement within the internal auditory canal, affecting the facial nerve on the left side more than the right, contributing to the auditory fullness on the left side. Cranial nerve involvement occurs in about a quarter of patients with neurosyphilis [15]. It is typically associated with syphilitic meningitis, as seen in this patient [16]. The facial nerve is one of the most commonly affected in neurosyphilis manifesting as syphilitic basilar meningitis [17].

## Conclusions

Neurosyphilis is rare but can have serious consequences if not identified and treated promptly. Diagnosis presents a challenge due to its status as a “great imitator” owing to a lack of specificity in its clinical presentation, especially in HIV-negative patients. However, it is important to recognize the potential for neurosyphilis in HIV-negative patients, particularly where other risk factors are present. Obtaining a thorough social history and recognizing recent trends in the epidemiology of neurosyphilis can help guide the course of clinical investigation. Physicians should readily suspect and test for neurosyphilis when indicated by sufficient risk factors since it is easy to miss given the nonspecific symptoms. This provides the opportunity for timely treatment and reduces the possibility of progression to late neurosyphilis and permanent neurological sequelae. They should also be aware of the gaps in sensitivity and specificity of various diagnostic and auxiliary measures for neurosyphilis, and use a combination of approaches to explore neurosyphilis as a possible diagnosis to best capture all available evidence.
